# Claims on Ready-to-Eat Cereals: Are Those With Claims Healthier?

**DOI:** 10.3389/fnut.2021.770489

**Published:** 2021-11-26

**Authors:** María Parra-Murillo, Caitlin M. Lowery, Luis F. Gómez, Mercedes Mora-Plazas, Lindsey Smith Taillie, Francesca R. Dillman Carpentier

**Affiliations:** ^1^Facultad de Medicina, Pontificia Universidad Javeriana, Bogota, Colombia; ^2^Department of Nutrition, Gillings School of Global Public Health, University of North Carolina, Chapel Hill, NC, United States; ^3^Departamento de Nutrición Humana, Facultad de Medicina, Universidad Nacional de Colombia, Bogotá, Colombia; ^4^Carolina Population Center, University of North Carolina, Chapel Hill, NC, United States; ^5^Hussman School of Journalism and Media, University of North Carolina at Chapel Hill, Chapel Hill, NC, United States

**Keywords:** marketing claims, breakfast cereal, claims, cereal bars, ready-to-eat food, marketing and advertising, critical nutrient content

## Abstract

**Background:** The use of advertising content strategies that suggest consuming a product will confer nutrient- and health-related benefits influences household food purchasing decisions, which increases consumption of energy-dense, nutrient-poor products. We examined the presence of marketing claims regarding nutrient content, health and nature in ready-to-eat (RTE) cereal packages in relation to the products' nutritional quality.

**Methods:** A cross-sectional content analysis was conducted on 178 RTE cereal packages available in the six largest supermarket chains in four Colombian cities from August to November 2018. The nutritional quality of products was assessed through the nutrient profile model established by the Chilean Law of Food Labeling and Advertising law.

**Results:** All products sampled exceeded the regulation threshold for at least one nutrient of concern (e.g., high-in calories and/or sugar). The majority (66.3%) of packages had claims related to nature, 57.3% had nutrient-content claims, and 15.7% had health benefit or risk avoidance claims. Most products with nature, nutrient-content, and health claims were high in energy (99.2, 98.0, and 92.9%, respectively) and sugar (88.1, 87.3, and 92.9%, respectively).

**Conclusion:** RTE cereal products offered in major Colombian supermarket chains are heavily marketed using nutrition- and nature-related claims. Nearly all products with claims are high in energy and sugar, despite the messages conveyed by the claims to consumers. Results support the implementation of mandatory regulations restricting claims on food and beverage products high in nutrients of concern.

## Introduction

Ready-To-Eat (RTE) cereals, as with Ultra-Processed Foods (UPF), are attractive to many consumers because of their taste, price, availability, shelf life, and ready-to-eat nature (1, 2). Such products are often considered highly appealing, are packaged in large servings, and are promoted through marketing messages that claim or imply that purchasing or consuming the product will confer specific benefits, such as improved health or social status (3). However, various studies that have assessed the impact of RTE cereals on dietary intakes and human health show that despite the supposed health benefits (4–8), the majority of these products are highly processed and high-in some nutrients like (4) sugar, sodium and unhealthy saturated fats (9). Nutrients frequently associated with consumption on increased dietary intake and weight gain (10), which in turn relate to an increased risk of overweight and obesity, abdominal obesity, all-cause mortality, metabolic syndrome, and depression in adults (9, 11).

This is a matter of concern if we consider the UPF has been particularly pronounced in Latin America and the Caribbean (12–14). In Colombia, a recent study found that adolescents and urban residents were the leading consumers of UPF, particularly snacks, processed cereals, dairy drinks and junk food (15). According to Euromonitor, in Colombia alone, the daily calorie intake per capita of RTE cereals has increased by 15.2% from 2009 to 2017 (16).

Marketing strategies may contribute to increased purchase and intake of UPF by creating a “health halo” effect, where consumers view a positive claim about health, nutrition, or nature and attribute that positive attribute to the whole product, perceiving the product to be healthier overall (17). Health claims include the suggestion of health benefits or reduced health risk from consuming the product (e.g., heart-healthy), nutrient-content claims highlight the presence of nutrients associated with positive health outcomes (e.g., high in fiber) (18), and nature claims identify a product or ingredient as natural (e.g., all-natural). Marketing claims related to nature can additionally reflect a valued aspect of one's identity, and so consumers may be predisposed to view foods with these claims as better choices (19, 20). Most consumers make their purchasing decisions in a matter of seconds (21), and marketing claims related to health, nutrients, and nature often influence their purchasing behavior (22, 23). As a result, these types of claims may increase sales of certain types of foods over others within a given category (24, 25).

Research around the world has revealed that health-related claims are widely used with products identified as unhealthy or with a high content of critical nutrients, such as sugar, fat, or sodium, according to the nutritional guidelines of regional or country-specific health organizations (26–28). For example, in Australia, 18.3% of nutrient content claims appeared on products that did not meet nutrient profile scoring criteria (29). Studies in Canada and Costa Rica have found that the proportion of sugar-sweetened milk drinks, breakfast cereals, and beverages with and without added sugar has a higher proportion of nutrition and health claims compared to other food categories (24, 30). In New Zealand, 65% of “less healthy” cereals had nutrition claims and 17% had health claims (31).

Breakfast cereals are among the foods most often marketed to children (32, 33). Products marketed to children, including RTE cereals, have a high number of health-related claims, even though many products with those claims do not meet national dietary guidelines or recommendations (34, 35). Yet, little is known about the types and prevalence of health, nutrient, and nature claims on RTE cereals in Colombia, specifically, or how the presence of these claims varies based on the product's nutritional profile. A better understanding of this issue is needed to inform policy actions aimed at reducing the prevalence of obesity and obesity-related diseases in Colombia through discouraging consumption of ultra-processed cereals (36). Therefore, the objective of this study is to examine the proportion of marketing claims about health, nutrient content, and nature-related found on RTE cereal packages offered at the most important supermarkets in the country as a function of the nutritional quality of the products.

## Methods

### Study Design

This study was developed through non-probabilistic purposive sampling of RTE cereal packages. A content analysis of marketing claims on the front of RTE cereals was combined with an analysis of the nutritional profile of each product based on Nutrition Facts Panel data.

The Institutional Review Board of the Pontificia Universidad Javeriana School of Medicine approved this study. An agreement with each supermarket chain was signed prior to data collection, which permitted photographs to be taken in the stores for study purposes.

### Data Collection

Product packaging images and data were collected cross-sectionally for all types of RTE cereals offered in the 33 supermarkets of the six largest grocery stores chains in the four largest cities in Colombia of highest sales according to Euromonitor, (Bogota, Medellin, Cali, and Barranquilla), during the months of August to November 2018. Agreements were signed with each of the six largest grocery store chains in the country to allow data collection. Data were collected on all products available for purchase during this time regardless of the product's country of origin. The data was collected using the Kanter photographic method used in previous studies in Colombia and Chile (37–40). Stores were in neighborhoods of high, middle, and low socioeconomic status across each city.

Data collectors were trained to take photographs of all sides of every RTE cereal package available in selected supermarkets. Photographs captured the following information: barcode, nutrition facts panel, ingredients, and portion size. Study data were collected and managed using REDCap (Research Electronic Data Capture) hosted at the University of North Carolina at Chapel Hill (41). If a product was available in multiple sizes, all packages were photographed and added to the database. If the content on the different-sized packages was the same, only one size of the product was retained for analysis. A total of 178 products were obtained in the Nutrition Fact Panel data from 2018.

### Coding Product Nutritional Quality

Accompanied by nutritionists, data collectors viewed photos and entered product and nutritional information into REDCap. For this study, RTE cereals were defined as products that include plain or blended flakes, puffed grains, processed grains, and fruit and flake mixes with or without other ingredients (42, 43); other cereals such as muesli and granola cereals were also included. RTE cereal do not include hot cereals, or wheat crackers because they must be cooked with hot water or hot milk to be ready for consumption (42, 43). In total, 178 RTE cereals were included in the sample, 128 breakfast cereals and 50 cereal bars. Products were classified as breakfast cereals or cereal bars according to their nutritional content based on the previous definition or the description provided by the manufacturer on the package (40).

The nutritional quality of RTE cereals was determined according to the 2019 implementation of the Chilean Nutrient Profile Model (CNPM) for solids (44). This model is one of the most common systems used or considered to identify products high in nutrients of concern, including added sugar, sodium, and saturated fat, as well as energy, in Latin America (40, 45, 46). A product qualifies for regulation under the 2019 CNPM if the product exceeds at least one of the following thresholds: (1) total sugar >10 g per 100 g; (2) saturated fat > 4 g per 100 g; (3) sodium > 400 mg per 100 g; or (4) total energy > 275 kcal per 100 g (44). Total sugars include free sugars and sugars that naturally occur in dairy products and intact fruit and vegetables (44). Products without *added* sugar, sodium, or saturated fat (e.g., plain milk without additives or 100% fruit juice) are exempt from the regulation. For this study, if a product exceeded the CNPM threshold for sugars, sodium, saturated fats, or calories, that product was coded as being in “high in” for being above the specific regulation threshold and it was coded as “not high in” for any threshold not exceeded.

Then, we calculated the proportion of products that included non-caloric sweeteners (NCS). The Pan American Health Organization Nutrient Profile Model includes NCS because habitual consumption of sweet-tasting foods (with or without sugar) increases the intake of sweet foods and beverages, including those containing sugar (47). This is especially important in children because consumption at an early age determines lifelong intake habits (47). Furthermore, NCS use in product reformulation has increased significantly in recent years (46, 48). Therefore, products with any amount of NCS were coded as “with NCS” (products that contained no NCS were coded as “without NCS”).

### Coding of Health, Nutrient-Content, and Nature-Related Claims

**Figure 1:**
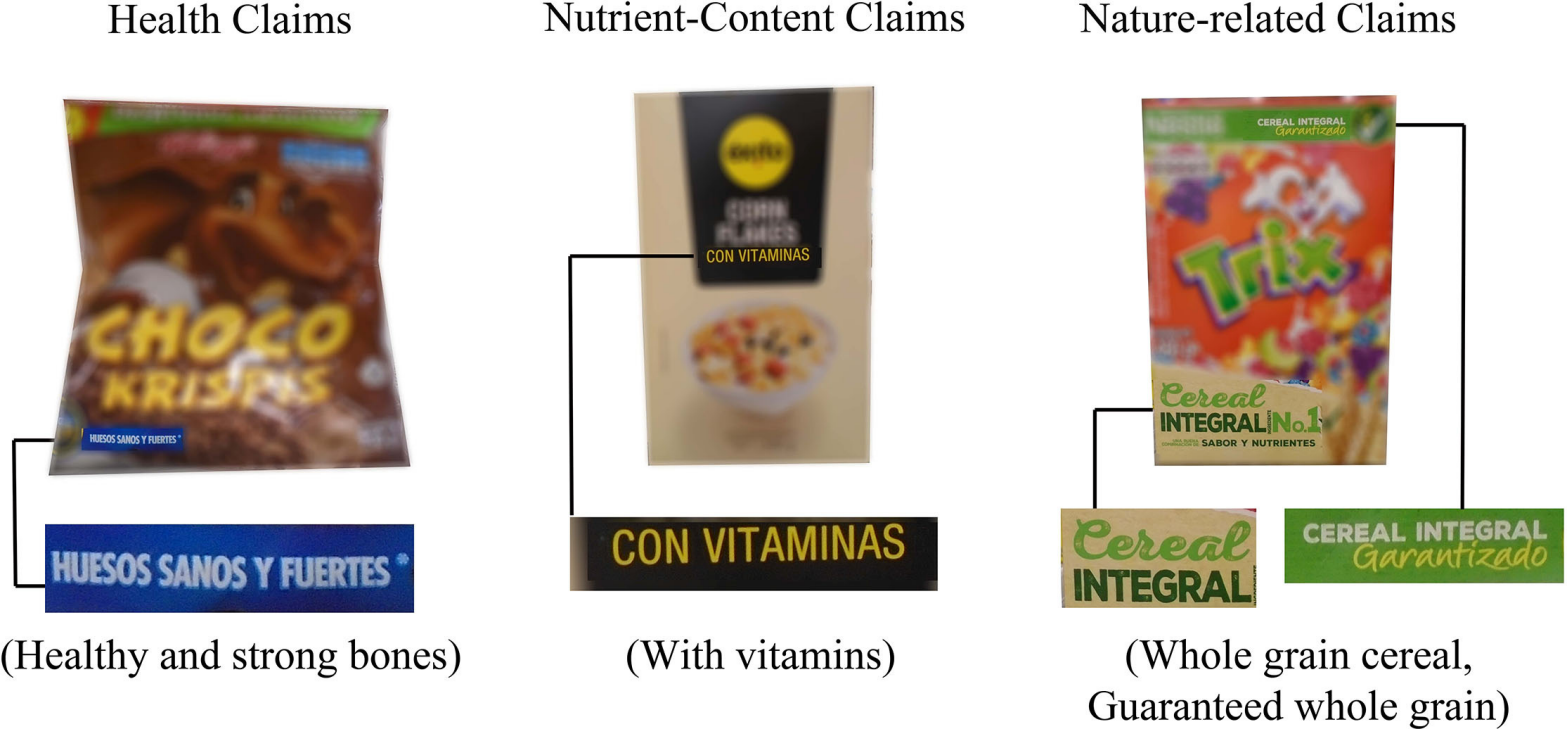
Examples of each of the types of claims found on the products.

A codebook adapted from a previous study on nutrient content claims on fruit drinks was used (49). Some of the claims overlap with those currently regulated by the FDA as health and nutrient content claims. Health claims were defined as any front-of-package (FOP) statements that expressly, or by implication, relate any substance in the product to a disease or health-related condition, for example, a claim that the cereal helps lower cholesterol or is heart-healthy (49). Nutrient-content claims were defined as any information displayed on the FOP that identifies a specific nutrient or ingredient as being present or absent in the product, such as being a good source of fiber or having no added sugar (49). Other claims that are not currently regulated by the FDA but were included in the study because of their importance in influencing future regulatory policy are “nature-related” claims (49, 50). Nature-related claims were defined as any statement that expressly, or by implication, suggests the product is natural, minimally processed, not artificial, organic, environmentally friendly, or produced sustainably (49, 51). For example, “whole grains” were classified as “nature-related” because they could be perceived as not having been significantly altered from their original chemical or biological state, without necessarily being associated with a health benefit or a specific nutrient (see Figure 1).

Specific claims that did not frequently appear on the studied products were grouped into an “Other” category by claim type. For example, in the subcategory “Text that mentions any other nutrient claims not already listed above,” we grouped the categories: low in calories, low in total fat, light, low in cholesterol, low in sugars, high in protein, good source of iron and gluten-free. This category also includes general nutrition-related statements like “Nutritivo” (Nutritious), “Desayunos nutritivos” (Nutritious breakfast) y “Sabor y nutrientes” (Taste and nutrients). In the subcategory “Text that mentions any other health claims not already listed above,” claims included “Fit,” “El placer de vivir bien” (The pleasure of well-being) y “Mantente fuerte” (Stay strong). Lastly, in the subcategory of “Text that mentions any other natural claims not already listed above,” we include the following categories: fresh or straight from the farm, organic and not containing GMOs. Sample claims include “Sans huile de palm” (Palm oil-free) and “Sin saborizantes ni colorantes artificiales” (No artificial flavors or colors); in addition, statements about nut or legume content were included in this category.

The entire sample of RTE cereal packages was double-coded independently by the first (M.P.) and third (L.F.G.) authors of this study, who used a standardized codebook to classify front-of-package (FOP) claims by claim type. Coders were trained on identifying claims on cookie packages. Agreement between coders was high, but in 9% of cases, disagreements were resolved by jointly reviewing the packages. In a few cases (0.3%), a nutritionist (M.M-P.) acted as judge and made a final decision. Given that the types of claims collected in the codebook were too numerous to be presented individually, the claims collected by the codebook were condensed into the categories described in Table 1 for analysis.

**Table 1:** Common claims displayed in Ready-To-Eat cereal packages (*n* = 178).

**Type of claims**	***n***	**%**
Nutrient-content claims	102	57.3
Good source of vitamins or minerals	64	35.9
Good source of calcium	14	7.8
Good source of fiber	14	7.9
Any other nutrient claim not already listed above	60	33.7
Health claims	28	15.7
Health claim about bone health	4	2.2
Health claim about digestion	19	10.7
Any other health claims not already listed above	3	1.7
Nature-related claims	118	66.3
Wholesome	39	21.9
Simple natural or unprocessed	40	22.5
Fruits and vegetables	71	39.9
Environmentally friendly or sustainable	16	8.9
Any other natural claims not already listed above	44	24.7

### Statistical Analysis

Data were analyzed using IBM SPSS Statistics (52) and Rstudio (53). Descriptive statistics (e.g., frequencies, medians) were used to describe the sample based on the amount of each critical nutrient in the product per 100 g and whether the product exceeded any regulation threshold in accordance with the 2019 CNPM. Cramer's V test was used to identify the strength of the relationship between high levels of critical nutrients between RTE cereal products with and without claims. For all analyses, a *p* ≤ 0.05 was considered significant.

## Results

All the analyzed RTE cereal packages (*n* = 178) were high in at least one of the nutrients considered in the CNPM, for more information on the nutritional quality of the products, see Supplementary Table 1. Additionally, 91% (*n* = 162) of these products carried a nature, nutrition, or health-related claim. The most common claims on the products were nature-related claims, carried by 66.3% (118) of the sample, including fruit claims (in Spanish: *con frutos rojos*/with red berries) and claims related to (lack of) processing (in Spanish: *con granos de maiz de or*í*gen natural*/with natural corn grains). Nutrient-content claims were also common, with 57.3% (102) of products containing claims like “good source of vitamins and minerals” (in Spanish: *8 vitaminas y 2 minerales*/8 vitamins and 2 minerals). Health claims were less common but appeared on 15.7% of products and included claims like “feel good about your digestion” (in Spanish: *siéntete bien con tu digestión*) (see Table 1).

Table 2 shows the number and percentage of products featuring at least one nutrient-content, health, or nature-related claims that were above the CNPM thresholds for saturated fat, sodium, sugars, and energy. Nearly all products with at least one claim were high in energy (98.7%), ranging from 92.9% of products with a health claim to 99.2% of products with a nature-related claim. Similarly, most products with claims were high in sugar (88.8%). While the percentage of products with claims that exceed the saturated fat and sodium thresholds was lower, 29% of products with claims were high in saturated fat and 24.7% were high in sodium. Cramer's V was used to analyze the strength of the relationship between high levels of critical nutrients and products with and without claims; however, although two of the variables (Health claims vs. High-In energy and Any Claim vs. High-in Sodium) obtained a significant *p*-value, Cramer's V does not reach the expected levels to be considered as even a moderate association. For this reason, it is not considered that there is a relationship between the variables. for more information about the comparison between products with and without claims (see Supplementary Table 2).

**Table 2:** Percentage of Ready-to-Eat cereal packages with claims that are high in critical nutrients.

**Claims**	***n***	**High-in energy**			**High-in saturated fat**			**High-in sodium**			**High-in sugar**			**High-in (Any)**	
		***N***	**%**	**Cramer's V**	***n***	**%**	**Cramer's V**	***n***	**%**	**Cramer's V**	***n***	**%**	**Cramer's V**	***n***	**%**
Nutrient-content claims	102	100	98.0	0.092	26	25.5	0.024	27	26.5	0.013	89	87.3	0.055	102	100
Health claims	28	26	92.9	0.247^**^	–	–	–	–	–	–	26	92.9	0.056	28	100
Nature-related claims	118	117	99.2	0.037	30	25.4	0.031	28	23.7	0.102	104	88.1	0.028	118	100
Any claim	162	160	98.8	0.034	47	28.4	0.144	40	24.7	0.163^*^	144	88.9	0.013	162	100

*Information obtained from 178 products. Due to the small sample size, we have not presented the proportions for cells with n <10. “High-in” per 100 g defined as: energy > 275 kcal, saturated fats > 4 g, sodium > 400 mg, sugars > 10 g*.

* *p < 0.05*;

** *p < 0.001. Cramer's V statistics were not been calculated in High-in (Any) because all products exceed the established limits in at least one critical nutrient, therefore it is a constant*.

In addition, more than a third of the products that had any claim contained NCS. Table 3 shows the percentage of products with claims containing NCS. According to Cramer's V the percentage of products with claims was not statistically significantly different from the percentage without claims with respect to the presence of NCS.

**Table 3:** Percentage of Ready-To-Eat cereal packages with claims that have NCS.

**Claims**	***n***	**Non-caloric sweeteners**		
		***n***	**%**	**Cramer's V**
Nutrient-content claims	102	37	36.3	0.049
Health claims	28	10	35.7	0.013
Nature-related claims	118	46	39.0	0.139
Any claim	162	57	35.2	0.061

Information obtained from 178 products. ^*^p < 0.05; ^**^p < 0.001.

## Discussion

This study provides an overview of the nutritional quality of RTE cereals offered in the principal Colombian supermarket chains in 2018, according to the presence or absence of health, nutrient-content, and natural claims. In general, all the analyzed cereals exceeded the regulation thresholds under the CNPM in at least one critical nutrient. This should be a cause for concern since 91% of the products analyzed contained some type of claim and the prevalence of critical nutrients seems to have increased. A previous study in Colombia found that 48.2% of cereal products were considered unhealthy according to international nutritional profile model standards (40).

In this study, the explicit textual claims most frequently displayed were related to alleged natural properties and benefits. In the case of natural claims, the most frequent claim pertained to flavor or fruit content. These results are consistent with previous studies in which flavor claims and nutritional claims are frequently used (49, 54). Similarly, previous studies have pointed to explicit references to flavor, texture, and fun as features that promote brand appeal by emphasizing and reinforcing sweet taste qualities (55). Although less frequent, the presence of claims like “wholesome,” “fresh” or “straight from the farm,” “simple,” “natural” or “unprocessed,” “no artificial flavors or colors” and “with nuts” or “with peanuts” were also common natural claims. Brands may use this marketing strategy to appear healthier or more sustainable (56) and to appeal to consumers who consider themselves “healthy eaters” and/or “environmentalists”(20). The true prevalence of nature-related claims may be underrepresented, as this study focused on textual claims and did not evaluate implicit marketing strategies, such as images of fruit or nature on the cereal bar and breakfast cereal packaging.

On the other hand, in the nutrient-content category, claims regarding vitamins and micronutrients (minerals) were the most frequent. These results are similar to those of previous studies conducted in the United Kingdom and Guatemala, where micronutrient and nutrient/ingredient claims were the most common type of claim on breakfast cereals (57, 58). In many cases, these claims were accompanied by the number of vitamins or minerals in the product, without specifying which vitamin or mineral they referred to. Many of these products are aimed at children and their parents. Parents may be motivated to purchase items for their children that contain “positive” ingredients like vitamins, minerals or fiber, but they may be less likely to look for and avoid products high in critical nutrients (56). In this sense, the presence of nutritional claims as a food marketing strategy (56) becomes a problem because the information with which they could contrast the messages is not clear and they are unable to know how nutritious it is on their own (59).

Similarly, parents tend to discard or overlook ingredients with which they are unfamiliar. Many of them are unaware of the presence of NCS in foods and do not regulate whether their children consume them (48). This is concerning given that the habitual consumption of sweet-tasting increases the intake of sweet foods and beverages, including those containing sugar (47). In the case of RTE cereals, previous studies have found that consumers of RTE cereals have a higher total daily intake of sugars than those who do not have breakfast with RTE cereals (60, 61). This may have important implications for NCS consumption because reformulation with NCS has emerged recently as an industry strategy to avoid “high-in sugar” warning labels. Although the effects of these substances are not yet definitive and more research is needed, it is believed that increased exposure to products with NCS may promote a greater liking for sweet foods, including those containing sugar (48). As mentioned earlier, this is especially important in children because consumption at an early age determines lifelong intake habits (47).

Except for the high-in energy density and presence or absence of health claims, no statistical differences were found between critical nutrients and NCS. This finding can be in part explained by the low reformulation of the food industry, in the period in which the information for this study was collected. For instance, a study that assessed the best-selling brands offered in Colombia between 2016 and 2018 found that only the beverage products reduced added sugar content (39). This could suggest that the industry did not have the incentive to use claims to promote reformulated products.

In comparison to natural and nutrient-content claims, health claims were less frequent. This aligns with findings from previous studies conducted in Mexico, Ecuador, Guatemala, and New Zealand (31, 35, 62). One possible explanation for this finding is that consumer associations may be more likely to sue for misleading or inaccurate health claims (63) however, more research is needed. Unlike nutrient-content and natural claims, health claims assume a supposed causal relationship between the consumption of the product and its effect on health, which is harder for a product manufacturer to demonstrate to a regulatory entity.

The finding that there was no statistically significant difference in the healthfulness of products with claims and without claims (in terms of critical nutrient or NCS content) provides evidence that while marketing claims suggest that products with claims are healthy (implicitly or explicitly), they are actually no healthier than products without claims. Claims are likely to mislead consumers into believing that these products are healthier than those without claims, when in fact they are not and may even be higher in one or more critical nutrients. These findings support the need for government intervention to protect its citizens' right to food and health, by implementing public policies that regulate marketing strategies, especially for products marketed to children, and developing a warning labeling system that can be easily understood by the majority of the population (64, 65).

### Strengths and Limitations

To our knowledge, this is the first Colombian study to characterize health, nutrition, and environmental claims in food products, using information obtained from the largest supermarket chains. Data analyzed for this study were obtained from photographs taken in major Colombian supermarkets in 2018, rather than from online databases that may not have up-to-date packaging images and may not accurately represent product availability in Colombia. In this study, data collectors took photographs of all RTE cereals available in outlets from six supermarket chains in four major cities in Colombia. All packages were independently double-coded by two study authors.

Several limitations can be identified in this study. First, we did not analyze implicit or indirect claims such as images of persons engaging in physical activities or famous people which may have important influence on acquiring and consuming a product. It should be noted that the lack of evaluation of implicit marketing strategies like pictures might underrepresent natural claims due to the presence of fruits and nature images on the imagery of the front of the RTE cereal packages. Second, the cross-sectional design of this study does not allow us to assess trends over time between nutritional quality of RTE cereal products in Colombia and the presence or absence of health, nutrition, and natural-related claims. Third, because the FDA has not regulated natural or environmentally related claims, these differences can be confusing. Sometimes they represent the level of processing in their biological or chemical characteristics and therefore could be considered “natural.” However, others are related to environmental responsibility. Although it is important to regulate both, it is useful to keep in mind that some are oriented to the level of processing and others are not (49, 50, 66).

Finally, future research should consider implicit claims and how consumers understand each of the claims, especially parents, who in most cases are responsible for the nutrition of their children and adolescents. Likewise, future studies should collect data at multiple time points and evaluate how the presence of these types of claims has varied over time.

## Conclusions

Given the prevalence of nutrient-content, health and nature-related claims on cereal products high in nutrients of concern, there is a need for the Colombian Congress and Government to implement policies restricting the use of claims on food and beverage products high-in critical nutrients, including clear definitions of the types of claims and considering those that have not been regulated to date.

## Data Availability Statement

The raw data supporting the conclusions of this article will be made available by the authors, without undue reservation.

## Author Contributions

LG, MM-P, and MP-M contributed to conception and design of the study. MP-M and LG organized the database. MP-M performed the statistical analysis. MP-M and CL wrote the first draft of the manuscript. LG, LT, FD, and MM-P wrote sections of the manuscript. All authors contributed to manuscript revision, read, and approved the submitted version.

## Funding

This research was funded by Bloomberg Philanthropies (Subward number # 5103721). The funder had no role in the study design, data collection, analysis or interpretation.

## Conflict of Interest

The authors declare that the research was conducted in the absence of any commercial or financial relationships that could be construed as a potential conflict of interest.

## Publisher's Note

All claims expressed in this article are solely those of the authors and do not necessarily represent those of their affiliated organizations, or those of the publisher, the editors and the reviewers. Any product that may be evaluated in this article, or claim that may be made by its manufacturer, is not guaranteed or endorsed by the publisher.

## References

[1] Poti JM Braga B Qin B. Ultra-processed food intake and obesity: what really matters for health-processing or nutrient content? Curr Obes Rep. (2017) 6:420–31. 10.1007/s13679-017-0285-4 29071481 PMC5787353

[2] Adams J Hofman K Moubarac JC Thow AM. Public health response to ultra-processed food and drinks. BMJ. (2020) 369:m2391. 10.1136/bmj.m2391 32591348 PMC7318879

[3] Anker T. Truth in Marketing: A Theory of Claim-Evidence Relations. 1st ed. Routledge, editor. London: Routledge Focus (2016).

[4] Perron J Pomerleau S Gagnon P Gilbert-Moreau J Lemieux S Plante C. Assessing nutritional value of ready-to-eat breakfast cereals in the province of Quebec (Canada): a study from the Food Quality Observatory. Public Health Nutr. (2021) 24:2397–404. 10.1017/S1368980021001361 33843558 PMC10195458

[5] Harris JL Schwartz MB Ustjanauskas A Ohri-Vachaspati P Brownell KD. Effects of serving high-sugar cereals on children's breakfast-eating behavior. Pediatrics. (2011) 127:71–6. 10.1542/peds.2010-0864 21149436

[6] Montenegro-Bethancourt G Vossenaar M Kuijper LD Doak CM Solomons NW. Ready-to-eat cereals are key sources of selected micronutrients among schoolchildren from public and private elementary schools in Quetzaltenango, Guatemala. Nutr Res. (2009) 29:335–42. 10.1016/j.nutres.2009.05.003 19555815

[7] Albertson AM Affenito SG Bauserman R Holschuh NM Eldridge AL Barton BA. The relationship of ready-to-eat cereal consumption to nutrient intake, blood lipids, and body mass index of children as they age through adolescence. J Am Diet Assoc. (2009) 109:1557–65. 10.1016/j.jada.2009.06.363 19699835

[8] Kosti RI Panagiotakos DB Zampelas A. Ready-to-eat cereals and the burden of obesity in the context of their nutritional contribution: are all ready-to-eat cereals equally healthy? A systematic review. Nutrs Res Rev. (2021) 23:314–22. 10.1017/S095442241000020X 20819244

[9] Lane MM Davis JA Beattie S Gómez-Donoso C Loughman A O'Neil A. Ultraprocessed food and chronic noncommunicable diseases: a systematic review and meta-analysis of 43 observational studie*s*. Obes Rev. (2020) 22:e13146. 10.1111/obr.13146 33167080

[10] Hall KD Ayuketah A Brychta R Cai H Cassimatis T Chen KY. Ultra-processed diets cause excess calorie intake and weight gain: an inpatient randomized controlled trial of *ad libitum* food intake. Cell Metab. (2019) 30:226. 10.31232/osf.io/w3zh2 31269427 PMC7959109

[11] Angelino D Rosi A Dall'asta M Pellegrini N Martini D. Evaluation of the nutritional quality of breakfast cereals sold on the italian market: The food labelling of italian products (flip) study. Nutrients. (2019) 11:2827. 10.3390/nu11112827 31752290 PMC6893738

[12] Popkin BM Corvalan C Grummer-Strawn LM. Dynamics of the double burden of malnutrition and the changing nutrition reality. Lancet. (2020) 395:65–74. 10.1016/S0140-6736(19)32497-3 31852602 PMC7179702

[13] Vandevijvere S Jaacks LM Monteiro CA Moubarac J Girling-Butcher M Lee AC. Global trends in ultraprocessed food and drink product sales and their association with adult body mass index trajectories. Obes Rev. (2019) 20:10–9. 10.1111/obr.12860 31099480

[14] Baker P Machado P Santos T Sievert K Backholer K Hadjikakou M. Ultra-processed foods and the nutrition transition: Global, regional and national trends, food systems transformations and political economy drivers. Obes Rev. (2020) 21:e13126. 10.1111/obr.13126 32761763

[15] Khandpur N Cediel G Obando DA Jaime PC Parra DC. Sociodemographic factors associated with the consumption of ultra-processed foods in Colombia. Rev Saude Publica. (2020) 54:1–13. 32049210 10.11606/s1518-8787.2020054001176 PMC7006913

[16] Euromonitor International. Breakfast Cereals in Colombia. (2019). Available online at: https://www.euromonitor.com/breakfast-cereals-in-colombia/report (accessed January 15, 2021).

[17] Acton RB Hammond D. Do manufacturer “nutrient claims” influence the efficacy of mandated front-of-package labels? Public Health Nutr. (2018) 21:3354–9. 10.1017/S1368980018002550 30345943 PMC10260817

[18] Martini D Del Bo' C Serafini M Porrini M Pellegrini N Angelino D. Breakfast Cereals Carrying Fibre-Related Claims: Do They Have a Better Nutritional Composition Than Those without Such Claims? Results from the Food Labelling of Italian Products (FLIP) Study. Foods. (2021) 10:2225. 10.3390/foods10092225 34574336 PMC8467444

[19] Schuldt JP Muller D Schwarz N. The “Fair Trade” effect. Soc Psychol Personal Sci. (2012) 3:581–9. 10.1177/1948550611431643

[20] Schuldt JP. Does green mean healthy? nutrition label color affects perceptions of healthfulness. Health Commun. (2013) 28:814–21. 10.1080/10410236.2012.725270 23444895

[21] Milosavljevic M Koch C Rangel A. Consumers can make decisions in as little as a third of a second. Judg Decis Making. (2011) 6:520–30.

[22] Gorton D Mhurchu CN Bramley D Dixon R. Interpretation of two nutrition content claims: a New Zealand survey. Aust N Z J Public Health. (2010) 34:57–62. 10.1111/j.1753-6405.2010.00474.x 20920106

[23] Ni Mhurchu C Gorton D. Nutrition labels and claims in New Zealand and Australia: a review of use and understanding. Aust N Z J Public Health. (2007) 31:105–12. 10.1111/j.1753-6405.2007.00026.x 17460999

[24] Franco-Arellano B Bernstein JT Norsen S Schermel A L'Abbé MR. Assessing nutrition and other claims on food labels: a repeated cross-sectional analysis of the Canadian food supply. BMC Nutr. (2017) 3:1–16. 10.1186/s40795-017-0192-9 32153852 PMC7050703

[25] Rodrigues VM Rayner M Fernandes AC De Oliveira RC Da Costa Proença RP Fiates GMR. Comparison of the nutritional content of products, with and without nutrient claims, targeted at children in Brazil. Br J Nutr. (2016) 115:2047–56. 10.1017/S0007114516001021 27040439

[26] Maschkowski G Hartmann M Hoffmann J. Health-related on-pack communication and nutritional value of ready-to-eat breakfast cereals evaluated against five nutrient profiling schemes. BMC Public Health. (2014) 14:1–11. 10.1186/1471-2458-14-1178 25407599 PMC4246564

[27] Stoltze FM Reyes M Taillie LS Correa T Corvalan C Carpentier FD. Prevalence of health and nutrient content marketing strategies on breakfast cereal packages before and after a countrywide marketing and labeling regulation: a focus on chile. Curr Dev Nutr. (2020) 4:1723. 10.1093/cdn/nzaa064_013

[28] Mclean R Hoek J Hedderley D. Effects of alternative label formats on choice of high-and low-sodium products in a New Zealand population sample. Public Health Nutr. (2011) 15:783–91. 10.1017/S1368980011003508 22281127

[29] Sussman RL McMahon AT Neale EP. An audit of the nutrition and health claims on breakfast cereals in supermarkets in the illawarra region of Australia. Nutrients. (2019) 11:1604. 10.3390/nu11071604 31311152 PMC6683094

[30] Gamboa-Gamboa T Blanco-Metzler A Vandevijvere S Ramirez-Zea M Kroker-Lobos MF. Nutritional content according to the presence of front of package marketing strategies: the case of ultra-processed snack food products purchased in costa rica. Nutrients. (2019) 11:1–14. 10.3390/nu11112738 31726678 PMC6893602

[31] Devi A Eyles H Rayner M Ni Mhurchu C Swinburn B Lonsdale-Cooper E. Nutritional quality, labelling and promotion of breakfast cereals on the New Zealand market. Appetite. (2014) 81:253–60. 10.1016/j.appet.2014.06.019 24953195

[32] Cairns G Angus K Hastings G Caraher M. Systematic reviews of the evidence on the nature, extent and effects of food marketing to children. A retrospective summary. Appetite. (2013) 62:209–15. 10.1016/j.appet.2012.04.017 22561190

[33] Castetbon K Harris JL Schwartz MB. Purchases of ready-to-eat cereals vary across US household sociodemographic categories according to nutritional value and advertising targets. Public Health Nutr. 15:1456–65. 10.1017/S1368980011003065 22152703

[34] Statista Market Forecast. Breakfast Cereals - Colombia. (2020). Available online at: https://www-statista-com.ezproxy.javeriana.edu.co/outlook/cmo/food/bread-cereal-products/breakfast-cereals/colombia (accessed May 10, 2021).

[35] Garcia AL Ronquillo JD Morillo-Santander G Mazariegos CV Lopez-Donado L Vargas-Garcia EJ. Sugar content and nutritional quality of child orientated ready to eat cereals and yoghurts in the UK and Latin America; does food policy matter? Nutrients. (2020) 12:1–11. 10.3390/nu12030856 32210128 PMC7146401

[36] Taillie LS Hall MG Popkin BM Ng SW Murukutla N. Experimental studies of front-of-package nutrient warning labels on sugar-sweetened beverages and ultra-processed foods: a scoping review. Nutrients. (2020) 12:569. 10.3390/nu12020569 32098363 PMC7071470

[37] Kanter R Reyes M Swinburn B Vandevijvere S Corvalán C. The food supply prior to the implementation of the chilean law of food labeling and advertising. Nutrients. (2018) 11:52. 10.3390/nu11010052 30597842 PMC6356190

[38] Kanter R Reyes M Corvalań C. Photographic methods for measuring packaged food and beverage products in supermarkets. Curr Dev Nutr. (2017) 1:e001016. 10.3945/cdn.117.001016 29955678 PMC5998779

[39] Lowery CM Mora-Plazas M Gómez LF Popkin B Taillie LS. Reformulation of packaged foods and beverages in the Colombian food supply. Nutrients. (2020) 12:3260. 10.3390/nu12113260 33114419 PMC7692620

[40] Mora-Plazas M Gómez LF Miles DR Parra DC Taillie LS. Nutrition quality of packaged foods in Bogotá, Colombia: a comparison of two nutrient profile models. Nutrients. (2019) 11:1–13. 10.3390/nu11051011 31060219 PMC6567873

[41] Harris PA Taylor R Thielke R Payne J Gonzalez N Conde JG. Research electronic data capture (REDCap)-a metadata-driven methodology and workflow process for providing translational research informatics support. J Biomed Inform. (2009) 42:377–81. 10.1016/j.jbi.2008.08.010 18929686 PMC2700030

[42] Australian government's Healthy Food Partnership. Ready-to-Eat Breakfast Cereal. (2016). Available online at: https://www1.health.gov.au/internet/main/publishing.nsf/Content/rte-cereal (accessed March 12, 2020).

[43] FAST R. Manufacturing Technology of Ready-to-Eat Cereals. Breakf Cereal How They Are Made. 2nd ed. Eagan, MN: Cereals and Grains Association (2000) p. 17–54.

[44] Ministerio de salud - Subsecretaria de salud pública. Ley 20.606 Sobre composición nutricional de los alimentos y su publicidad. Santiago de Chile, Chile: Ministerio de Salud (2012).

[45] Corvalán C Reyes M Garmendia ML Uauy R. Structural responses to the obesity and non-communicable diseases epidemic: update on the Chilean law of food labelling and advertising. Obes Rev. (2019) 20:367–74. 10.1111/obr.12802 30549191

[46] Correa T Fierro C Reyes M Dillman Carpentier FR Taillie LS Corvalan C. Responses to the Chilean law of food labeling and advertising: exploring knowledge, perceptions and behaviors of mothers of young children. Int J Behav Nutr Phys Act. (2019) 16:21. 10.1186/s12966-019-0781-x 30760273 PMC6375144

[47] Pan American Health Organization. Pan American Health Organization Nutrient Profile Model. Washington: Pan American Health Organization (2016) p. 1–38.

[48] Martínez X Zapata Y Pinto V Cornejo C Elbers M van der Graaf M. Intake of non-nutritive sweeteners in chilean children after enforcement of a new food labeling law that regulates added sugar content in processed foods. Nutrients. (2020) 12:1–14. 10.3390/nu12061594 32485840 PMC7352803

[49] Duffy EW Hall MG Dillman Carpentier FR Musicus AA Meyer ML Rimm E. Nutrition claims on fruit drinks are inconsistent indicators of nutritional profile: a content analysis of fruit drinks purchased by households with young children. J Acad Nutr Diet. (2021) 121:36–46.e4. 10.1016/j.jand.2020.08.009 32978105 PMC7752796

[50] The Food and Drug Administration. Use of the Term Natural on Food Labeling | FDA. (2016). Available online at: https://www.fda.gov/food/food-labeling-nutrition/use-term-natural-food-labeling (accessed July 1, 2021).

[51] Dunckel M Schweihofer J Kuschel A. Natural and Organic Label Claims - Agriculture. MSU Extension Agriculture. Meridian Charter Township, MI (2020) p. 1.

[52] IBM Corp. IBM SPSS Statistics for Windows. Armonk, NY: IBM Corp (2020).

[53] RStudio Team. RStudio: Integrated Development Environment for R. Boston, MA: RStudio Team (2020).

[54] Kim K Cheong Y Zheng L. The current practices in food advertising: the usage and effectiveness of different advertising claims. Int J Advert. (2009) 28:527–53. 10.2501/S0265048709200722

[55] McGinnis JM Gootman JA Kraak VI. Food Marketing to Children and Youth : Threat or Opportunity? (2006). Available online at: http://search.ebscohost.com/login.aspx?direct=true&db=e000xww&AN=159570&site=eds-live (accessed March 12, 2020).

[56] Hughner RS Maher JK. Factors that influence parental food purchases for children: implications for dietary health. J Mark Manag. (2006) 22:929–54. 10.1362/026725706778935600

[57] García AL Morillo-Santander G Parrett A Mutoro AN. Confused health and nutrition claims in food marketing to children could adversely affect food choice and increase risk of obesity. Arch Dis Child. (2019) 104:541–6. 10.1136/archdischild-2018-315870 30530844

[58] Soo J Letona P Chacon V Barnoya J Roberto CA. Nutritional quality and child-oriented marketing of breakfast cereals in Guatemala. Int J Obes. (2016) 40:39–44. 26293234 10.1038/ijo.2015.161

[59] Dixon H Scully M Wakefield M Kelly B Chapman K Donovan R. Parent's responses to nutrient claims and sports celebrity endorsements on energy-dense and nutrient-poor foods: an experimental study. Public Health Nutr. (2010) 14:1071–9. 10.1017/S1368980010003691 21306666

[60] Priebe MG Mcmonagle JR. Effects of ready-to-eat-cereals on key nutritional and health outcomes: a systematic review. PLoS ONE. (2016) 11:e0164931. 10.1371/journal.pone.0164931 27749919 PMC5066953

[61] Williams PG. The benefits of breakfast cereal consumption: a systematic review of the evidence base 1-4. Adv Nutr. (2014) 5:636–73. 10.3945/an.114.006247 25225349 PMC4188247

[62] Nieto C Rincon-Gallardo Patiño S Tolentino-Mayo L Carriedo A Barquera S. Characterization of breakfast cereals available in the mexican market: sodium and sugar content. Nutrients. (2017) 9:884. 10.3390/nu9080884 28813010 PMC5579677

[63] Nestle M Ludwig DS. Front-of-package food labels: public health or propaganda? JAMA. (2010) 303:771–2. 10.1001/jama.2010.179 20179287

[64] Guarnizo-Peralta D. Sin Reglas ni controles Regulación de la publicidad de alimentos y bebidas dirigidas a menores de edad. Centro de Estudios de Derecho Justicia y Sociedad D, editor. Bogotá: Centro de Estudios de Derecho, Justicia y Sociedad, Dejusticia (2017). p. 1–34.

[65] Mediano Stoltze F Busey E Taillie LS Dillman Carpentier FR. Impact of warning labels on reducing health halo effects of nutrient content claims on breakfast cereal packages: a mixed-measures experiment. Appetite. (2021) 163:105229. 10.1016/j.appet.2021.105229 33789168

[66] Federal Register. Use of the Term “Natural” in the Labeling of Human Food Products; Request for Information and Comments. Washington, WA: Federal Register (2016) p. 5.

