# Supplementary material for: Claims on Ready-to-Eat Cereals: Are Those With Claims Healthier?
Source: Front Nutr. 2021 Nov 26;8:770489. doi: 10.3389/fnut.2021.770489 (PMC8662936; doi:10.3389/fnut.2021.770489)
Supplement: Supplementary file 1 [file Table_1.docx]

Supplementary Material

# Supplementary Tables

Supplementary Table 1. Mean, median, IQR and percentage of Ready-To-Eat cereals according to have or not have a high contain of critical nutrients (n=178)

| **Critical nutrients** | | **n** | **%** | **Mean** | **Median per 100g** | **IQR** |
| --- | --- | --- | --- | --- | --- | --- |
| **Energy (kcal/100g)** | **Not high-in** | 2 | 1.1 | - | - | - |
|  | **High-in** | 176 | 98.9 | 405.1 | 400.0 | 66.6 |
| **Saturated fat** | **Not high-in** | 131 | 73.6 | 0.7 | 0.0 | 1.6 |
|  | **High-in** | 47 | 26.4 | 7.0 | 6.7 | 3.3 |
| **Sodium** | **Not high-in** | 130 | 73.0 | 215.5 | 226.7 | 224.6 |
|  | **High-in** | 48 | 27.0 | 528.4 | 500.0 | 100.0 |
| **Sugar** | **Not high-in** | 20 | 11.2 | 7.9 | 10.0 | 4.4 |
|  | **High-in** | 158 | 88.8 | 27.9 | 30.0 | 13.3 |

Information obtained from 178 products. Due to the small sample size, we have not presented the statistics for cells with n<10. “High-in” per 100g defined as: energy >275g, saturated fats >4g, sodium >400mg, sugars >10g.
