# Supplementary material for: Claims on Ready-to-Eat Cereals: Are Those With Claims Healthier?
Source: Front Nutr. 2021 Nov 26;8:770489. doi: 10.3389/fnut.2021.770489 (PMC8662936; doi:10.3389/fnut.2021.770489)
Supplement: Supplementary file 2 [file Table_2.docx]

Supplementary Table 2. Nutritional content and presence of claims in Ready-To-Eat cereals.

| **Critical nutrients** | | **Nutrient-Content Claims** | | | | **Health Claims** | | | | **Nature-related Claims** | | | |
| --- | --- | --- | --- | --- | --- | --- | --- | --- | --- | --- | --- | --- | --- |
|  |  | **Without Claims** | | **With Claims** | | **Without Claims** | | **With Claims** | | **Without Claims** | | **With Claims** | |
|  |  | **n** | **%** | **n** | **%** | **n** | **%** | **n** | **%** | **n** | **%** | **n** | **%** |
| **Energy** | **High-in** | 76 | 42.9 | 100 | 57.1 | 150* | 85.1 | 26* | 14.9 | 59 | 33.7 | 117 | 66.3 |
|  | **Not high-in** | 0 | - | 2 | - | 0 | - | 2 | - | 1 | - | 1 | - |
| **Saturated fat** | **High-in** | 21 | 44.7 | 26 | 55.3 | 41 | 87.2 | 6 | - | 17 | 36.2 | 30 | 63.8 |
|  | **Not high-in** | 55 | 42.0 | 76 | 58.0 | 109 | 83.2 | 22 | 16.8 | 43 | 32.8 | 88 | 67.2 |
| **Sodium** | **High-in** | 21 | 43.8 | 27 | 56.3 | 42 | 87.5 | 6 | - | 20 | 41.7 | 28 | 58.3 |
|  | **Not high-in** | 55 | 42.3 | 75 | 57.7 | 108 | 83.1 | 22 | 16.9 | 40 | 30.8 | 90 | 69.2 |
| **Sugar** | **High-in** | 69 | 43.7 | 89 | 56.3 | 132 | 83.5 | 26 | 16.5 | 54 | 34.2 | 104 | 65.8 |
|  | **Not high-in** | 7 | - | 13 | 65.0 | 18 | 90.0 | 2 | - | 6 | - | 14 | 70.0 |

Information obtained from 178 products. Due to the small sample size, we have not presented the statistics for cells with n<10. “High-in” per 100g defined as: energy >275g, saturated fats >4g, sodium >400mg, sugars >10g. *P-value < 0.05
