# Supplementary material for: Claims on Ready-to-Eat Cereals: Are Those With Claims Healthier?
Source: Front Nutr. 2021 Nov 26;8:770489. doi: 10.3389/fnut.2021.770489 (PMC8662936; doi:10.3389/fnut.2021.770489)
Supplement: Supplementary file 3 [file Table_3.docx]

Supplementary Table 3. Mean, median, IQR and percentage of Ready-To-Eat cereals according to have or not have a high contain of critical nutrients separated by cereal bars and breakfast cereals (n=178).

| **Critical nutrients** | | **Breakfast Cereals (n=128)** | | | | **Cereal Bars (n=50)** | | | |
| --- | --- | --- | --- | --- | --- | --- | --- | --- | --- |
|  |  | **n** | **%** | **Median per 100g** | **IQR** | **n** | **%** | **Median per 100g** | **IQR** |
| **Energy (kcal/100g)** | High-in | 126 | 98.4 | 370.9 | 33.3 | 50 | 100.0 | 425.3 | 61.1 |
|  | Not high-in | 2 | - | - | - | 0 | - | - | - |
| **Saturated Fat (g)** | High-in | 16 | 12.5 | 6.7 | 1.7 | 31 | 62.0 | 7.0 | 5.2 |
|  | Not high-in | 112 | 87.5 | 0.0 | 1.0 | 19 | 38.0 | 1.6 | 2.3 |
| **Sodium (mg)** | High-in | 43 | 33.6 | 500.0 | 108.3 | 5 | - | - | - |
|  | Not high-in | 85 | 66.4 | 283.3 | 193.3 | 45 | 90.0 | 152.2 | 95.7 |
| **Sugar (g)** | High-in | 114 | 89.1 | 30.0 | 13.3 | 44 | 88.0 | 25.6 | 11.85 |
|  | Not high-in | 14 | 10.9 | 10.0 | 0.0 | 6 | - | - | - |

Information obtained from 178 products. Due to the small sample size, we have not presented the statistics for cells with n<10. “High-in” per 100g defined as: energy >275g, saturated fats >4g, sodium >400mg, sugars >10g.
