# Supplementary material for: Claims on Ready-to-Eat Cereals: Are Those With Claims Healthier?
Source: Front Nutr. 2021 Nov 26;8:770489. doi: 10.3389/fnut.2021.770489 (PMC8662936; doi:10.3389/fnut.2021.770489)
Supplement: Supplementary file 4 [file Table_4.docx]

Supplementary Table 4. Percentage of Ready-To-Eat cereal packages with claims that have Non-Caloric Sweeteners (NCS) separated by cereal bars and breakfast cereals.

| **Non-Caloric Sweeteners** | | **n** | **Nutrient-Content Claims** | | **Health Claims** | | **Nature-related Claims** | |
| --- | --- | --- | --- | --- | --- | --- | --- | --- |
|  |  |  | **n** | **%** | **n** | **%** | **n** | **%** |
| **Without NCS** | **Breakfast Cereals** | 105 | 61 | 56.5 | 16 | 14.8 | 62 | 57.4 |
|  | **Cereal Bars** | 12 | 4 | - | 2 | - | 10 | 83.3 |
| **With NCS** | **Breakfast Cereals** | 23 | 18 | 78.3 | 7 | - | 14 | 60.9 |
|  | **Cereal Bars** | 38 | 19 | 50.0 | 3 | - | 32 | 84.2 |

Information obtained from 178 products. Due to the small sample size, we have not presented the statistics for cells with n<10. “High-in” per 100g defined as: energy >275g, saturated fats >4g, sodium >400mg, sugars >10g.
